# SOCIAL ISOLATION AND DELAYED DEMENTIA DIAGNOSIS AMONG US OLDER ADULTS

**DOI:** 10.1093/geroni/igad104.0698

**Published:** 2023-12-21

**Authors:** Min Hee Kim, Ruijia Chen, Grisell Diaz-Ramirez, John Boscardin, M Maria Glymour

**Affiliations:** University of California San Francisco, San Francisco, California, United States; University of California San Francisco, San Francisco, California, United States; University of California San Francisco, San Francisco, California, United States; University of California San Francisco, San Francisco, California, United States; University of California San Francisco, San Francisco, California, United States

## Abstract

Delayed dementia diagnosis may exacerbate dementia symptoms and reduce opportunities for care arrangements, further harming older adults’ health and well-being. Social isolation may increase the risk of delayed dementia diagnosis via a lack of support that prevents seeking help and accessing primary and specialty care for diagnosis. We investigated the association between social isolation and missed/delayed dementia diagnosis using the Health and Retirement Study (2004-2018) linked with the Medicare Claims record. We defined social isolation as having 3 or more affirmative responses to 6 questions about living arrangements, family/friend contact, and social participation. We defined missed/delayed diagnosis as having no clinical diagnosis documented in Medicare in the presence of an algorithm-defined dementia diagnosis in the survey; or having dementia diagnosed more than 3 years after meeting the survey-based algorithmic criterion for dementia. We fitted logistic regression models, adjusting for sociodemographic and health characteristics. Among 2,417 people in our sample (mean age 82.4(SD 5.85); 60% women), 54.7% of older adults experienced social isolation at the time of survey-based dementia onset, 41.5% had missed/delayed diagnosis, and 22.1% died with no dementia diagnosis in their Medicare record. Preliminary results indicate that social isolation was not associated with increased odds of missed/delayed diagnosis (OR 1.09 95% CI 0.90, 1.32). Our findings suggest that social isolation may not be a major driver of healthcare access and system factors pertinent to clinical documentation of dementia. To corroborate findings, we plan to refine our models (e.g., accounting for attrition and selection).

